# Handgrip strength cutoff for cardiometabolic risk index among Colombian children and adolescents: The FUPRECOL Study

**DOI:** 10.1038/srep42622

**Published:** 2017-02-14

**Authors:** Robinson Ramírez-Vélez, Jhonatan Camilo Peña-Ibagon, Javier Martínez-Torres, Alejandra Tordecilla-Sanders, Jorge Enrique Correa-Bautista, Felipe Lobelo, Antonio García-Hermoso

**Affiliations:** 1 Centro de Estudios para la Medición de la Actividad Física «CEMA». Escuela de Medicina y Ciencias de la Salud, Universidad del Rosario, Bogotá D.C., Colombia; 2 Hubert Department of Global Health, Rollins School of Public Health, Emory University, Atlanta, GA, USA; 3 Laboratorio de Ciencias de la Actividad Física, el Deporte y la Salud, Facultad de Ciencias Médicas, Universidad de Santiago de Chile, USACH, Santiago, Chile

## Abstract

Evidence shows an association between muscular strength (MS) and health among young people, however low muscular strength cut points for the detection of high metabolic risk in Latin-American populations are scarce. The aim of this study was twofold: to explore potential age- and sex-specific thresholds of MS, for optimal cardiometabolic risk categorization among Colombian children and adolescents; and to investigate whether cardiometabolic risk differed by MS group by applying the receiver operating characteristic curve (ROC) cut point. MS was estimated by using a handle dynamometer on 1,950 children and adolescents from Colombia, using MS relative to weight (handgrip strength/body mass). A metabolic risk score was computed from the following components: waist circumference, triglycerides, HDL-c, glucose, and systolic and diastolic blood pressure. ROC analysis showed a significant discriminatory accuracy of MS in identifying the low/high metabolic risk in children and adolescents and in both genders. In children, the handgrip strength/body mass levels for a low metabolic risk were 0.359 and 0.376 in girls and boys, respectively. In adolescents, these points were 0.440 and 0.447 in girls and boys, respectively. In conclusion, the results suggest an MS level relative to weight for having a low metabolic risk, which could be used to identify youths at risk.

Poor muscular strength (MS), as determined with the use of a handgrip (HG) dynamometer, is recognized as a marker of poor metabolic profile during adolescence^1^ and is associated with disease and mortality in adulthood^2^,^3^. Most current studies support an inverse relationship between low MS and cardiovascular disease risk factors in young people, generally expressing muscular strength in relative terms. Our group^4^ and other researchers^5^,^6^,^7^ have shown an independent and inverse association between low strength and cardiometabolic risk clustering among adolescents and adults. In addition, Ruiz *et al*.^8^ and Ortega *et al*.^1^ reported, in a systematic review, the relationship between MS and health outcomes such as lipid profile and glucose levels, particularly in overweight and obese children, respectively.

The HG strength test is a quick and easy-to-perform muscular fitness test that provides useful information about overall MS, and it could potentially be used in the clinical setting^9^,^10^. Clinical examinations and HG measurements are described in detail by Artero *et al*.^5^, Smith *et al*.^11^ and Ortega *et al*.^12^. The contribution of low MS to the progression of secondary sedentary behavior with aging and/or cardiometabolic risk factors (e.g. obesity, systemic low-grade inflammation, insulin resistance) is equally unequivocal, and recent national efforts to identify cut points or thresholds for lambda-mu-sigma (LMS) among young people^13^,^14^ will help clinicians to screen individuals at greatest risk^15^.

Previous studies have shown that there is a relationship between MS and cardiometabolic risk factors in young and adult populations. However, there is no consensus regarding the minimum MS level associated with a clustered cardiometabolic risk among youth in Latin America. Therefore, from a public health perspective, the inclusion of HG in health surveillance systems is clearly justifiable; schools may also be an ideal setting for monitoring the fitness of young people to identify those with poor MS^16^,^17^. In order to identify children and adolescents in whom low MS is a potential contributor to cardiometabolic risk factors, it is necessary to determine clinical screening strategies in young population. Thus, the aim of this study was twofold: to explore potential age- and sex-specific thresholds of MS, for optimal cardiometabolic risk categorization among Colombian children and adolescents aged 9 to 17.9 years; and to investigate whether cardiometabolic risk differed by MS group by applying the receiver operating characteristic curve (ROC) cut point.

## Methods

### Participants and study design

This is a secondary analysis of a cross-sectional study (the FUPRECOL study), published elsewhere^18^,^19^. The FUPRECOL study assessments were conducted during the 2014–2015 school year. The sample consisted of children and adolescents (boys *n* = 4,000 and girls *n* = 4,000) aged 9–17.9 years. In a subgroup of 2,775 schoolchildren, biomarker parameters were also assessed and a more exhaustive health and lifestyle assessment was carried out. From this subgroup, 1,950 schoolchildren (64.5% adolescents) showed valid data from the HG, anthropometric and blood parameter assessments, and were consequently used in this study. The schoolchildren were of low-middle socioeconomic status (SES, 1–3 defined by the Colombian government) from public elementary and high schools (grades 5 and 11) in the capital district of Bogota in a municipality in the Cundinamarca Department in the Andean region. A convenience sample of volunteers was included and grouped by sex and age with 1-year increments (a total of 9 groups). Power calculations were based on the mean HG from the first 150 participants in the ongoing data collection (range 25–35 kg), with a group SD of approximately 9.9 kg. The significance level was set to 0.05, and the required power was set to at least 0.80. The sample size was estimated to be approximately 80 to 100 participants by sex and age group. Exclusion factors included a clinical diagnosis of cardiovascular disease, diabetes mellitus 1 and 2, pregnancy, the use of alcohol or drugs and, in general, the presence of any disease not directly associated with nutrition. Exclusion from the study was made effective a posteriori, without the students being aware of it, to avoid any undesired situations.

The study was approved by the institutional review board for the use of human subject research in addition to the Rosario University Board (Code N° CEI-ABN026-000262). Potential subjects and their parents or guardian(s) were informed of the purpose, benefits, and potential risks of the study, and then provided written informed consent to participate. The protocol was in accordance with the latest revision of the Declaration of Helsinki (as revised in Hong Kong in 1989 and in Edinburgh, Scotland, in 2000) and current Colombian laws governing clinical research on human subjects (Resolution 008430/1993 of the Ministry of health).

### Procedures

HG was measured using a standard adjustable-handle Takei Digital Grip Strength Dynamometer, Model T.K.K.540^®^ (Takei Scientific Instruments Co., Ltd, Niigata, Japan). In accordance with predetermined protocols^20^, the dynamometer grip opening was adjusted to the subject’s hand size. The study participants had previously received brief instructions (verbal and demonstration) regarding measurement procedures. HG was measured with the subject in a standing position, with the shoulder adducted and neutrally rotated and arms parallel but not in contact with the body. Two trials were allowed with each limb and the average score was recorded as the peak grip strength (kg). Thus, the HG values presented here combine the results of left- and right-handed subjects, without considering hand dominance. Several studies suggest that links between MS and both physical function and health status are directly mediated by the proportion of MS relative to body weight. Also, there is substantial covariance between MS capacity and body weight. Therefore, to avoid the potential biasing effect of body weight on the estimation of MS, HG was adjusted for body weight in line with standard assumptions about morphologic effects as previous studies^21^,^22^ have suggested [i.e. (HG strength in kg)/(body weight in kg)]. This methodology was recently used in a similar large study in American adolescents^20^. HG measurements in a subsample (n = 229, similar in demographics and biological characteristics to the whole sample) were recorded to ensure reproducibility on the day of the study. The reproducibility of our data was R = 0.96. Intra-rater reliability was assessed by determining the intraclass correlation coefficient (0.98, CI 95% 0.97 to 0.99).

Anthropometric variables were measured by a Level 2 anthropometrist certified by the International Society for the Advancement of Kinanthropometry (ISAK), in accordance with the ISAK guidelines^23^, in the morning following an overnight fast, at the same time (7:00–10:00 a.m.). Body weight and height were measured with the subjects in their underwear and with no shoes, using electronic scales (Tanita^®^ BC544, Tokyo, Japan; TEM = 0.510%) and a mechanical stadiometer platform (Seca^®^ 274, Hamburg, Germany; TEM = 0.01%), respectively. The average of the two readings of weight and height was used to calculate body mass index (BMI) as weight (kg) divided by height squared (m^2^). Weight status was defined as having a BMI above the age- and sex-specific thresholds of the International Obesity Task Force (IOTF)^24^. Waist circumference was measured with the patient in the standing position without clothing at the midpoint level of the mid-axillary line between the 12^th^ rib head and the superior anterior iliac spine using a tape measure (Ohaus^®^ 8004-MA, New Jersey, USA; TEM = 0.86%). Hip circumference was taken at the largest point at the level of the greater trochanters, and thigh circumference was measured midway between the hip and knee (Ohaus^®^ 8004-MA, New Jersey, USA; TEM = 0.91%). All circumference measures were calculated as the average of three measurements.

In addition, percentage of body fat was assessed by bioelectrical impedance using a bipolar TANITA^®^ BF-689 floor scale (Arlington Heights, IL 60005, USA) and the results were expressed as percentage of body weight. Briefly, the subject stood with their feet slightly apart, and the instrument recorded impedance from foot to foot and subsequently percentage of body fat to the nearest 0.1% based on age, gender, height, and weight. TEM was 0.63 and the repeatability coefficient 0.98%. According to Kasvis *et al*.^25^, the bipolar BIA equipment has been shown to be reliable and valid because it includes prediction equations to estimate body fat percentage adjusted by age and gender in 5- to 17-year-old children. Validation tests and equations are available from the manufacturer’s website (http://www.tanita.com/en/bf-689/) or from the study conducted by Kasvis *et al*.^25^.

Sexual maturation was classified based on Tanner staging^26^, which uses self-reported puberty status to classify participants into stages I to V^27^. Each volunteer entered an isolated room where they categorized the development of their own genitalia (for boys), breasts (for girls), armpits (for boys), and pubic hair (for both genders) using a set of images exemplifying the various stages of sexual maturation. The reproducibility of our data reached *R* = 0.78.

### Biochemical assessments

Blood samples were collected between 6:00 and 8:00 am by two experienced pediatric phlebotomists after at least 12 hours of fasting. Before the extraction, fasting conditions were confirmed by the child and parents. Blood samples were obtained from an antecubital vein, and analyses were subsequently completed within 1 day from collection. The levels of triglycerides (TG), total cholesterol (TC), cholesterol linked to high-density lipoproteins (HDL-c), and glucose were measured using colorimetric enzymatic methods with the use of a Cardiochek analyzer. The fraction of cholesterol linked to low-density lipoproteins (LDL-c) was calculated using the Friedewald formula^28^. The precision performance of these assays was within the manufacturer’s specifications.

### Cardiometabolic risk assessment

We calculated a cardiometabolic risk index (CMRI) as the sum of the age and sex standardized scores of WC, TG, HDL-c, glucose, and systolic and diastolic blood pressure^29^. The HDL-c value was then multiplied by −1 as this is inversely related to cardiovascular risk. An age-adjusted continuous cardiometabolic risk score (composite z-score) was calculated for each participant as follows:


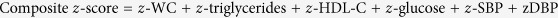


The components of the score were selected on the basis of the International Diabetes Federation^30^ and modified De Ferranti *et al*.^31^ definitions of metabolic syndrome. High risk was defined as ≥1 SD of this score. The higher the value in the CMRI, the higher the cardiovascular risk. All cutoff values were based on data about international schoolchildren^32^,^33^,^34^.

### Statistical analysis

Anthropometric, biochemical profiles and MS characteristics of the study sample are presented as means and standard deviations (SD). The normality of selected variables was verified using histograms and Q-Q plots. Differences were analyzed by two-way analysis of variance (ANOVA) *or* Chi-square test (X^2^) to explore sex and age differences. Cutoff values were derived mathematically from the ROC curves, using the point on the ROC curve with the lowest value for the formula: (1-sensitivity)^2^ + (1-specificity)^2^. The positive likelihood ratio LR (+) and the negative likelihood ratio LR (−) were used to analyze the potential diagnostic accuracy of the HG (kg)/body mass (kg) to discriminate between low and high CMRI. The area under the curve (AUC) and 95% confidence interval (CI) were calculated. The AUC represents the ability of the test to correctly classify children and adolescents with a low/high CMRI. The AUC values can range between 1 (perfect test) and 0.5 (worthless test). Finally, an ANOVA was used to investigate whether cardiometabolic risk differed by MS group by applying the ROC cut point in both gender and age groups. Data were analyzed with SPSS for Windows (SPSS, Chicago, Illinois, USA). A *p* value under 0.05 denoted statistical significance.

## Results

The 1,950 scholars included 691 boys (54.7% boys, 9 to 12.9 years old) and 1,259 girls (56.6% girls, 13.0 to 17.9 years old). Their mean age was 12.9 ± 2.3 years. Overall, boys had higher levels of hip circumference, body fat, and triglycerides than girls (p < 0.001), whereas girls had lower HG and normalized strength per body mass (p < 0.05). The prevalence of overweight and obesity was 26.3% and 9.8% in girl children, and 22.7% and 5.5% in adolescent girls, respectively (p < 0.05), according to the IOTF criteria (Table 1). In boy children, the prevalence of overweight and obesity was 18.3% and 10.9%, and it was 10.8% and 5.7% in adolescent boys, respectively (p < 0.05).

**Figure 1:**
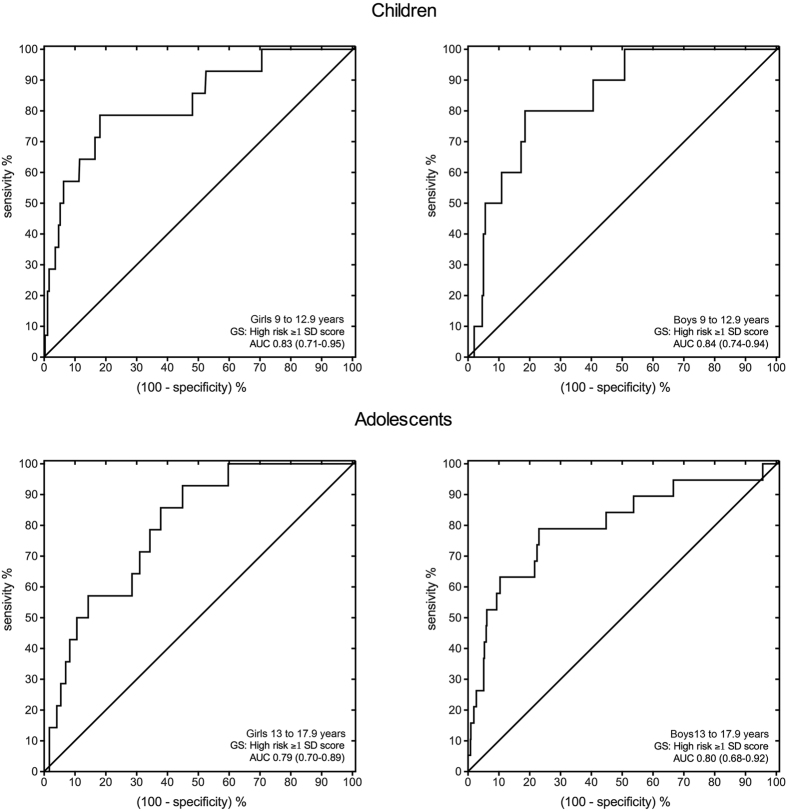
Receiver operating characteristic (ROC) curve of the normalized grip strength [measured as (grip strength in kg)/(body mass in kg)], to detect low CMRI in both sexes among children and adolescents. GS: gold standard; AUC: area under the curve (95% confidence interval).

ROC analyses showed a significant discriminatory accuracy in identifying the low/high CMRI in both gender and age groups (AUC = 0.83 (95%CI: 0.71–0.95), p < 0.001; boys AUC = 0.84 (95%CI: 0.74–0.94), p < 0.001; adolescent girls AUC = 0.79 (95%CI: 0.70–0.89), p < 0.001; boys AUC = 0.88 (95%CI: 0.68–0.92), p < 0.001). In children (9 to 12.9 years old), handgrip strength (kg)/body mass (kg) values at these points were 0.359 and 0.376 in girls and boys, respectively. In adolescents (13.0 to 17.9 years old), these points were 0.440 and 0.447 in girls and boys, respectively (Table 2 and Fig. 1).

Thresholds were determined as the corresponding normalized strength at the low/high CMRI, per sex and group categories. Group- and sex-specific thresholds for high strength and low strength are provided for children (Table 3) and adolescents (Table 4), with corresponding anthropometric and biochemical profile differences. In both groups, thresholds may, therefore, be used to categorize individuals into two categories of risk (i.e. low and high risk) on the combined basis of sex and age group and combined grip strength capacity. In children and adolescents, ANOVA showed that there were differences in BMI, all circumferences (waist, hip, and mid-upper arm), adiposity (body fat), and biochemical profile.

## Discussion

The present study builds on earlier findings about the relationship between MS and cardiovascular disease by examining the predictive ability of different cutoffs for MS in detecting children and adolescents at risk. Thus, our results describe pragmatic MS cut points reflecting differences in several cardiometabolic risk factors between young people above the thresholds (adequate/high level of MS) and those who did not (low level of MS).

Our results show that among children (9 to 12.9 years old), cutoffs for handgrip strength/body mass level associated with low metabolic risk were 0.359 and 0.376 in girls and boys, respectively. In adolescents (13 to 17.9 years old), these cut points were 0.440 and 0.447 in girls and boys, respectively. The objective of this study was similar to that of another recent study: to determine thresholds of muscular weakness for the prediction of cardiometabolic risk factors in a large cohort (n = 1,326) of adolescents^20^. In this study, Peterson *et al*. reported a high-risk threshold for boys (≤0.33) and girls (≤0.28), as well as an intermediate threshold (boys, >0.33 and ≤0.45; girls, >0.28 and ≤0.36). Our results are in agreement with those of Pederson *et al*., which included a composite score of normalized strength. Although absolute strength testing is certainly more specific for informing exercise prescription, it is not readily viable as a screening tool for a clinical setting.

The ROC curves generated for this study showed acceptable AUC and 95% confidence interval limits, suggesting that the resultant cut points were not due to chance (all AUC ≥ 0.79) and distinguished between children and adolescents at high cardiometabolic risk on the basis of HG relative to body weight. Our findings underscore the need for public health interventions to promote MS as a potentially effective strategy for lowering cardiovascular disease risk in later life. As suggested by the World Health Organization physical activity recommendations^35^, MS activities to enhance muscular fitness should be included at least three days a week.

The role of MS has been increasingly recognized in the prevention of chronic disease in adults^3^. Low MS in children and adolescents associated with poorer current cardiometabolic health predicts higher cardiovascular risk in adulthood^36^ and mortality from total and cardiovascular disease in adulthood^3^. Previous studies have shown that MS is inversely associated with metabolic risk^7^, principally in high-income countries and largely in Caucasian cohorts^21^. In Colombian schoolchildren of similar social status to the present sample from Bucaramanga city, Cohen *et al*.^7^ showed that poorer HG/body mass was associated with a worse metabolic risk profile; specifically, young people in the lowest strength quartile were three times more likely to have elevated metabolic risk than those in the highest strength quartile. In the present study, youths classified as having a “low level of MS” displayed significantly higher clustered cardiometabolic risk scores than those who reached the MS threshold. The exact mechanisms that explain the protective effect of MS on cardiometabolic risk in young populations have not yet been established. Steene-Johannessen *et al*.^6^ hypothesized that the apparent protective effect of MS in children and adolescents could be a function of puberty, however these authors also reported an association in prepubertal children and in both sexes.

There are some limitations to this study. The cross-sectional design prevents us from establishing a causal relationship. Also, the ROC analysis does not enable adjustments for potential confounders within the model, and data were not adjusted prior to ROC analysis. The thresholds were not applied to a different cohort of youths from the population that the ROC curves were originally generated to determine the usefulness of. Finally, the study participants were teenagers and hand size can be influenced by height, which could have an effect on MS values. However, we applied a standard protocol to avoid measurement errors. Nevertheless, the inclusion of a large number of subjects of the same age and the objective measurement of MS and biochemical parameters are notable strengths of this study. Also, the HG strength test is a simple, low-cost method of assessing MS and its use is urged at a population level.

## Conclusion

Our study identifies MS cut points associated with high cardiometabolic risk in youth. These cut points are the first to be based on the widely used HG assessment and provide a highly useful tool for classifying young populations at risk of cardiometabolic disease. Longitudinal studies are needed to establish the incidence of cardiovascular and metabolic diseases later in life based on MS levels during childhood and adolescence, especially in low-middle-income countries.

## Additional Information

**How to cite this article**: Ramírez-Vélez, R. *et al*. Handgrip strength cutoff for cardiometabolic risk index among Colombian children and adolescents: The FUPRECOL Study. *Sci. Rep.* **7**, 42622; doi: 10.1038/srep42622 (2017).

**Publisher's note:** Springer Nature remains neutral with regard to jurisdictional claims in published maps and institutional affiliations.

## Figures and Tables

**Table 1:** Characteristics of children and adolescents with anthropometric, biochemical profile and muscular strength.

	Children (9–12.9 years) n = 691		*p value*	Adolescents (13–17.9 years) n = 1259		*p value*
	Girls n = 378	Boys n = 313		Girls n = 713	Boys n = 546	
Age (years)	10.1 (0.7)	10.0 (0.8)	0.377	13.14 (0.8)	13.15 (0.8)	0.138
Height (cm)	139.4 (8.5)	138.5 (8.4)	<0.001	152.8 (6.7)	156.18 (9.9)	<0.001
Body mass (kg)	35.5 (8.0)	35.1 (8.2)	0.006	47.4 (8.6)	47.18 (10.0)	<0.001
Body mass index (kg/m^2^)	18.0 (2.7)	18.1 (2.8)	0.540	20.2 (2.8)	19.19 (2.8)	<0.001
Weight status n(%)^a^						
Underweight	69 (18.3)	24 (7.7)	0.027	101 (14.2)	90 (16.5)	<0.001
Normal	173 (45.6)	198 (63.1)		411 (57.6)	366 (67.0)	
Overweight	99 (26.3)	57 (18.3)		162 (22.7)	59 (10.8)	
Obesity	37 (9.8)	34 (10.9)		39 (5.5)	31 (5.7)	
Waist circumference (cm)	59.0 (6.8)	61.2 (7.3)	<0.001	64.0 (6.9)	65.32 (6.8)	0.605
Hip circumference (cm)	74.3 (7.7)	73.1 (7.7)	<0.001	85.0 (7.9)	81.41 (7.9)	<0.001
Body fat BIA (%)	24.6 (6.1)	16.2 (6.1)	<0.001	22.7 (6.2)	18.5 (6.6)	<0.001
Tanner stage n(%)^a^						
Pre-puberty	64 (16.9)	69 (22.1)	0.001	12 (1.7)	11 (2.0)	0.002
Puberty	312 (82.5)	239 (76.3)		672 (94.2)	498 (91.2)	
Post-puberty	2 (0.5)	5 (1.6)		29 (4.1)	37 (6.8)	
SBP (mmHg)	109.8 (14.0)	111.2 (13.7)	0.881	109.4 (12.2)	111.5 (14.1)	0,011
DBP (mmHg)	66.6 (8.4)	67.0 (87.9)	0.655	68.2 (8.3)	66.9 (9.0)	0.622
Total cholesterol (mg/dl)	153.7 (30.5)	153.9 (30.0)	0.632	147.7 (28.7)	138.8 (29.6)	<0.001
HDL cholesterol (mg/dl)	48.6 (13.2)	52.1 (12.5)	<0.001	47.5 (11.9)	46.6 (12.3)	0.003
LDL cholesterol (mg/dl)	88.2 (27.0)	86.8 (29.8)	0.908	84.2 (27.1)	82.6 (33.4)	0.006
Triglycerides (mg/dl)	97.4 (67.7)	85.8 (41.8)	0.006	93.9 (50.8)	84.1 (38.8)	0.001
Glucose (mg/dl)	83.3 (14.9)	85.4 (14.7)	0.144	81.5 (15.4)	81.5 (15.4)	0.028
CMRI	0.028 (0.52)	−0.027 (0.48)	0.198	−0.041 (0.48)	−0.018 (0.53)	0.216
Handgrip (kg)	14.9 (3.7)	15.3 (3.6)	0.001	21.1 (4.2)	24.2 (6.9)	<0.001
Handgrip (kg)/body mass (kg)	0.439 (0.08)	0.441 (0.08)	<0.001	0.453 (0.08)	0.521 (0.10)	<0.001

CMRI, cardiometabolic risk index.

**Table 2:** Cut-off between sensitivity and specificity for the normalized grip strength [measured as (grip strength in kg)/(body mass in kg)] to screen for CMRI by sex and group aged.

	Children (9–12.9 years) n = 691		Adolescents (13–17.9 years) n = 1259	
	Girls n = 378	Boys n = 713	Girls n = 713	Boys n = 546
AUC	0.83 (0.71–0.95)	0.84 (0.74–0.94)	0.79 (0.70–0.89)	0.80 (0.68–0.92)
Cut-off	0.359	0.376	0.440	0.447
J-Youden	0.61	0.62	0.48	0.56
Sensitivity	78.6	80.0	92.9	78.9
Specificity	81.9	81.5	55.1	77.0
Positive likelihood ratio	4.34	4.32	2.07	3.43
Negative likelihood ratio	0.26	0.25	0.13	0.27

**Table 3:** Sex thresholds for high and low normalized grip strength [measured as (grip strength in kg)/(body mass in kg)], with anthropometric, biochemical profile and CMRI among Colombian children 9 to 12.9 years old.

	Children					
	Girls (n = 378)			Boys (n = 313)		
	<0.359	≥0.359	*p value*	<0.376	≥0.376	*p value*
Body mass index (kg/m^2^)	20.4 (3.5)	17.9 (2.6)	<0.0001	21.0 (4.1)	17.7 (2.6)	<0.0001
Waist circumference (cm)	65.1 (7.7)	60.5 (6.5)	<0.0001	67.7 (9.5)	61.5 (6.6)	<0.0001
Hip circumference (cm)	79.2 (8.6)	75.4 (8.3)	<0.0001	78.4 (9.2)	73.4 (7.7)	<0.0001
Body fat BIA (%)	27.4 (6.5)	21.9 (5.6)	<0.0001	31.3 (8.2)	23.9 (7.4)	<0.0001
Systolic blood pressure (mmHg)	109.7 (14.8)	107.8 (14.5)	0.028	108.5 (13.4)	108.2 (15.5)	0.762
Diastolic blood pressure (mmHg)	66.2 (10.1)	66.4 (10.6)	0.759	66.7 (10.6)	66.6 (11.0)	0.882
Total cholesterol (mg/dl)	154.0 (28.5)	152.1 (29.4)	0.588	150.9 (28.5)	154.2 (30.8)	0.386
HDL cholesterol (mg/dl)	47.0 (12.6)	49.4 (13.3)	0.114	46.9 (11.3)	53.1 (13.2)	<0.0001
LDL cholesterol (mg/dl)	89.5 (27.6)	85.0 (26.2)	0.145	84.6 (25.4)	86.1 (29.8)	0.670
Triglycerides (mg/dl)	103.1 (52.1)	92.7 (41.0)	0.038	100.1 (51.7)	84.0 (42.8)	0.005
Glucose (mg/dl)	86.1 (15.6)	84.0 (15.1)	0.223	89.0 (16.7)	85.1 (16.5)	0.066

**Table 4:** Sex thresholds for high and low normalized grip strength [measured as (grip strength in kg)/(body mass in kg)], with anthropometric, biochemical profile and CMRI among Colombian adolescents 13 to 17.9 years old.

	Adolescents					
	Girls (n = 713)			Boys (n = 546)		
	<0.440	≥0.440	*p value*	<0.447	≥0.447	*p value*
Body index mass (kg/m^2^)	22.8 (3.4)	20.3 (2.6)	<0.0001	22.9 (4.1)	19.7 (2.6)	<0.0001
Waist circumference (cm)	70.1 (7.9)	65.2 (6.4)	<0.0001	72.7 (9.5)	67.7 (6.7)	<0.0001
Hip circumference (cm)	90.3 (8.3)	86.5 (6.8)	<0.0001	88.2 (9.8)	84.5 (8.4)	<0.0001
Body fat BIA (%)	27.4 (6.4)	23.5 (6.1)	<0.0001	21.0 (8.0)	13.8 (5.3)	<0.0001
Systolic blood pressure (mmHg)	111.9 (12.3)	109.7 (13.2)	<0.0001	115.1 (14.4)	113.9 (14.8)	0.176
Diastolic blood pressure (mmHg)	69.5 (9.8)	68.5 (9.7)	0.008	70.4 (9.1)	69.1 (10.8)	0.046
Total cholesterol (mg/dl)	150.2 (31.3)	147.1 (30.4)	0.131	141.8 (35.0)	131.3 (30.0)	0.001
HDL cholesterol (mg/dl)	45.6 (11.2)	48.0 (11.9)	<0.002	42.7 (12.5)	44.3 (10.9)	0.192
LDL cholesterol (mg/dl)	86.1 (27.5)	83.5 (31.6)	0.210	83.5 (35.2)	76.2 (35.1)	0.049
Triglycerides (mg/dl)	105.1 (64.1)	88.7 (35.5)	<0.0001	94.0 (45.5)	82.8 (33.0)	0.003
Glucose (mg/dl)	80.6 (16.4)	81.2 (16.8)	0.554	85.0 (13.5)	82.6 (16.0)	0.151
